# Budd-Chiari Syndrome Caused by TIPS Malposition: A Case Report

**DOI:** 10.1155/2014/267913

**Published:** 2014-04-13

**Authors:** A. S. Katkar, Anderson H. Kuo, S. Calle, K. Gangadhar, K. Chintapalli

**Affiliations:** ^1^Department of Radiology, University of Texas Health Science Center at San Antonio, 7703 Floyd Curl Drive, San Antonio, TX 78229-3900, USA; ^2^Department of Radiology, Hospital Universitario San Ignacio, Kra 7, No. 40-62, Bogotá, Colombia

## Abstract

Budd-Chiari syndrome refers to hepatic pathology secondary to diminished venous outflow, most commonly associated with venothrombotic disease. Clinically, patients with Budd-Chiari present with hepatomegaly, ascites, abdominal distension, and pain. On imaging, Budd-Chiari syndrome is hallmarked by occluded IVC and or hepatic veins, caudate lobe enlargement, heterogeneous liver enhancement, intrahepatic collaterals, and hypervascular nodules. Etiopathological factors for Budd-Chiari syndrome include several systemic thrombotic and nonthrombotic conditions that can cause venous outflow obstruction at hepatic veins and/or IVC. While the transjugular intrahepatic portosystemic shunt (TIPS) is used as a treatment option for Budd-Chiari syndrome, Budd-Chiari syndrome is not a well-known complication of TIPS procedure. We report a case of Budd-Chiari syndrome that occurred in a transplanted cirrhotic liver from malpositioned proximal portion of the TIPS in IVC causing occlusion of the ostia of hepatic veins which was subsequently diagnosed on contrast-enhanced CT.

## 1. Case Report

**Figure 1:**
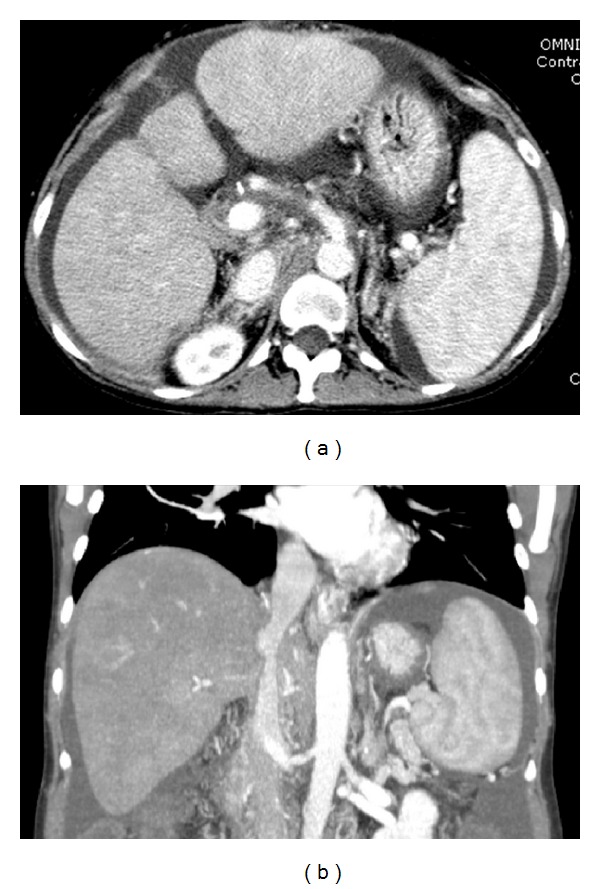
(a) Axial and (b) coronal contrast-enhanced CT of the upper abdomen obtained at admission shows a liver of cirrhotic morphology and nodular contours with mild diffuse heterogeneous enhancement. Splenomegaly, partly visualized ascites, and gastroesophageal varices are also noted. These findings were consistent with cirrhosis and sequela of portal hypertension. Numerous surgical clips about the porta hepatis represent changes of orthotopic liver transplantation.

**Figure 2:**
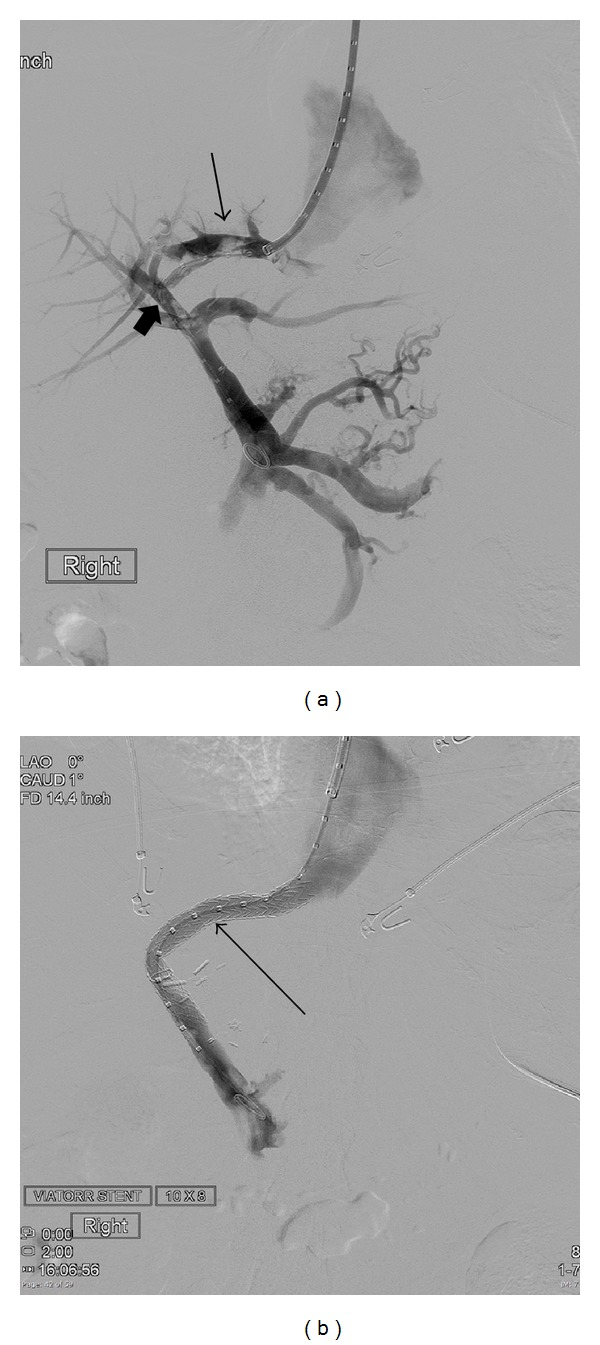
(a) Portovenogram performed during TIPS procedure shows a radioopaque graduated pigtail catheter in place, maintaining the tract created between the middle hepatic vein (*thin arrow*) and the inferior branch of the right portal vein (*thick arrow*). (b) Portovenogram with TIPS stent in place (*arrow*). The proximal uncovered portion was within the inferior branch of the right portal vein and the distal end was within the middle hepatic vein just before joining the inferior vena cava.

**Figure 3:**
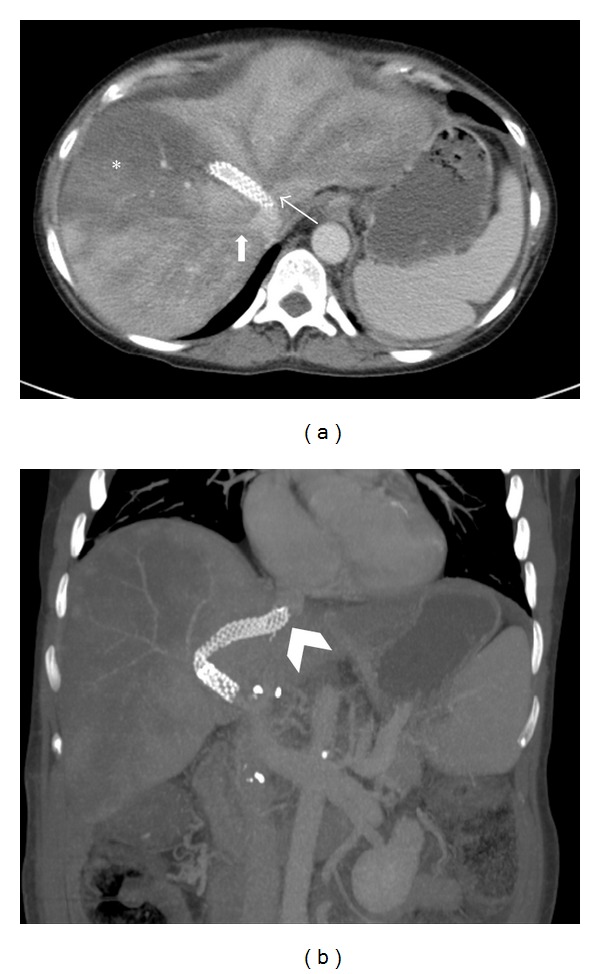
(a) Axial contrast-enhanced CT of the upper abdomen during the portal-venous phase demonstrates thrombosed left and middle hepatic veins (*thin arrows*). The proximal end of the TIPS stent is seen within the IVC which is occluding ostia of the left and middle hepatic veins (*black arrow*). The right hepatic vein is patent and adequately opacified with IV contrast (*thick arrow*). A wedge-shaped hypodense area in hepatic segments V and VIII (*asterisk*) representing hepatic infarction. (b) Coronal MIP image of the upper abdomen shows the position of the TIPS stent, communicating the right portal vein and the mid hepatic vein with its most cranial tip within the IVC (*arrowhead*).

**Figure 4:**
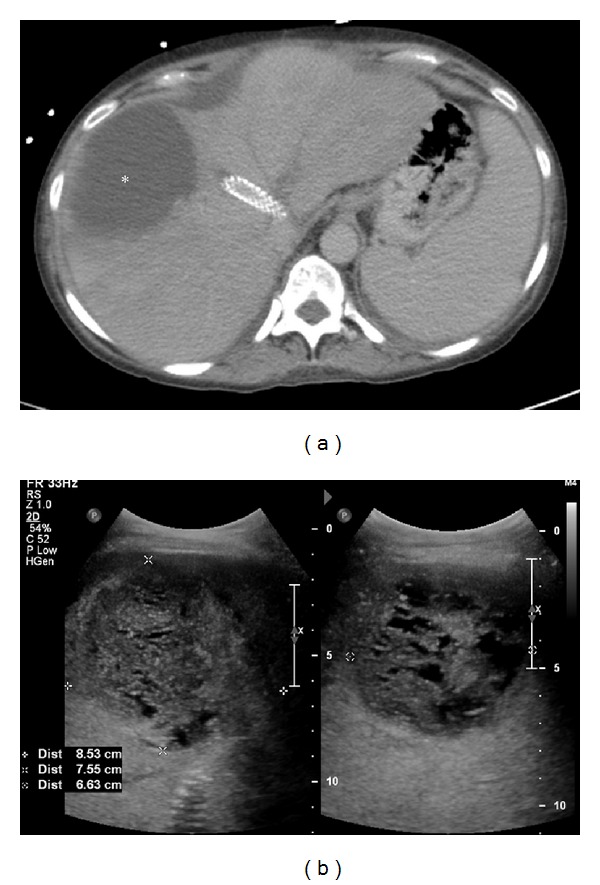
(a) Axial contrast-enhanced CT of the upper abdomen shows a well-defined nonenhancing hypodense area liquefaction necrosis of infarcted liver parenchyma in hepatic segments V and VIII (*asterisk*). Again noted is the hypodense material within the left hepatic vein, consistent with hepatic vein thrombosis. (b) Ultrasound of the right lobe of the liver demonstrates a heterogeneous collection that corresponds to hepatic infarction.

A 46-year-old female with medical history of cryptogenic cirrhosis, status postorthotopic liver transplantation done 9 years ago, presented to the tertiary care hospital with end-stage liver disease, hepatic encephalopathy, and abdominal distension secondary to recurrent ascites, requiring frequent paracentesis (Figure 1). Successful creation of TIPS was performed using 8 cm long and 1 cm wide Viatorr stent graft from the middle hepatic vein to the inferior branch of the right portal vein (Figure 2). Placement of the stent resulted in a decrease in the portosystemic gradient from 27 mmHg to 11 mmHg. There was subsequent resolution of the abdominal distension. Two weeks after TIPS placement, however, the patient returned to the hospital with fever, nausea, and right upper quadrant pain. Admission CT demonstrated thrombosed left and middle hepatic veins in heterogeneously enhancing enlarged liver with wedge-shaped hypoattenuating areas in hepatic segments V and VIII. Also noted was the TIPS stent terminating in the inferior vena cava overlapping the ostia of the left and middle hepatic veins and associated thrombosis of left and middle hepatic veins (Figure 3). Patient also underwent liver Doppler which showed tardus parvus waveforms in main and right hepatic artery suggestive of hepatic arterial stenosis. Ten days later, a follow-up CT of the abdomen and ultrasound was performed showing interval development of a fluid collection at the site of the previously documented liver hypoattenuation, consistent with hepatic abscess formation (Figure 4), which was later drained via percutaneous approach using ultrasound guidance.

## 2. Discussion

Josef Rösch and his coworkers first developed the TIPS procedure in the year 1969. The shunt was originally performed unintentionally during transjugular cholangiography when Rösch inadvertently entered the portal vein and realized the potential that this communication had to relieve complications of portal hypertension [1]. Subsequently, stents were implanted to maintain the patency within the shunt, initially using silicone-coated spring coil, and then later, metallic materials [2]. Initially used to treat refractory ascites and hemorrhage from esophageal varices secondary to portal hypertension, TIPS is currently an accepted therapy for other conditions such as hepatic hydrothorax, hepatorenal, hepatopulmonary, and Budd-Chiari syndromes [1].

Morbidity and mortality are usually low in TIPS procedure. Major complication rate is documented at less than 5% and mortality rate at less than 2% [3, 4]. Freedman et al. in reviewing literature categorized potential complications of TIPS into those related to (a) needle puncture and puncture site, (b) portal venous access, (c) portal venous cannulation/dilation, (d) stent positioning and thrombosis, (e) portosystemic shunting, and (f) contrast material [5]. Fatal procedural complications are usually due to intraperitoneal hemorrhage, laceration of the hepatic artery or portal vein, and right heart failure [1]. Deterioration of liver function and hepatic encephalopathy represent the most common complications following TIPS [1]. Migration of the stent represents less than 3% of the complications in that study [5]. Silva et al. in a retrospective analysis of 41 TIPS patients observed migration in 8% of the cases, with equal portions to the portal vein and the right atrium [6].

Budd-Chiari syndrome results from partial or complete hepatic venous outflow obstruction either at the level of the hepatic veins, IVC, or right atrium. Consequently, hepatic congestion ensues due to increased hepatic sinusoidal pressure, which in turn leads to portal hypertension and decreased liver perfusion. This process may ultimately progress to liver fibrosis and cirrhosis [7]. Clinically, this syndrome may have varying manifestations according to the extension and acuteness of the obstruction. Clinically, patients with Budd-Chiari present with hepatomegaly (90%), ascites (83%), abdominal distension (77%), and pain (61%). Symptoms usually include portal hypertension, ascites, and liver failure and may range from mild to fulminant in severity [7]. Budd-Chiari syndrome may occur due to intrinsic conditions such as prothrombotic hematologic disorders that predispose patients to blood clot formation. Other causes include metastatic invasion of the hepatic vein, IVC, or right atrium or extrinsic compression due to tumor formation within neighboring organs (e.g., kidney, liver, adrenal glands, etc.) [7].

Severe ascites, hepatomegaly and patchy irregular enhancement of the liver parenchyma are commonly seen acute findings on CT [8]. There is typically greater enhancement in the central portion of the liver with decreased peripheral enhancement and the thrombosed vessel shows hypoattenuation. As the syndrome progresses, subacute and chronic Budd-Chiari may demonstrate areas of hypoperfusion within the liver as well as morphologic changes and development of collateral vessels [7, 8]. Chronic Budd-Chiari syndrome displays changes similar to other long-stage fibrotic conditions of the liver, with the appearance of multiple regenerative nodules [8]. To our knowledge, TIPS is not considered as a well-known cause of Budd-Chiari syndrome. In the case that we are reporting, the proximal portion of the TIPS stent was malpositioned in IVC which secondarily occluded the drainage of the left hepatic vein and middle hepatic vein. The obstruction and resultant blood stasis were considered the precipitating factors for subsequent thrombus formation within the vessel and the onset of Budd-Chiari syndrome. Patient also had wedge-shaped hypodense areas in hepatic segments V and VIII indicating hepatic infarction which on follow-up ultrasound showed evidence of liquefaction necrosis and abscess formation. Hepatic infarction was likely the result of ischemia caused by hepatic arterial stenosis which was evident on Doppler study (not shown here).

The case that we are presenting is of particular interest because it establishes stronger cause and effect relationship of hepatic venous thrombosis (Budd-Chiari syndrome) and malpositioned TIPS stent occluding the ostia of hepatic veins, both of which are clearly evident on the CT scan.

Treatment for Budd-Chiari syndrome includes both medical and surgical options with the primary goal of resolving hepatic congestion and therefore preserving liver function. Patients with mild symptoms and minimal or no necrosis of the liver may be adequately managed medically with pharmacologic control of ascites with diuretics, anticoagulant therapy, and management of any underlying condition [7]. More severe cases may require shunt creation and liver transplantation.

## References

[1] Owen AR, Stanley AJ, Vijayananthan A, Moss JG (2009). The transjugular intrahepatic portosystemic shunt (TIPS). *Clinical Radiology*.

[2] Rossle M (2013). TIPS: 25years later. *Journal of Hepatology*.

[3] Ripamonti R, Ferral H, Alonzo M, Patel NH (2006). Transjugular intrahepatic portosystemic shunt-related complications and practical solutions. *Seminars in Interventional Radiology*.

[4] Sawhney R, Wall SD (1998). TIPS complications. *Techniques in Vascular and Interventional Radiology*.

[5] Freedman AM, Sanyal AJ, Tisnado J (1993). Complications of transjugular intrahepatic portosystemic shunt: a comprehensive review.. *Radiographics*.

[6] Silva RF, Arroyo PC, Duca WJ (2004). Complications following transjugular intrahepatic portosystemic shunt: a retrospective analysis. *Transplantation Proceedings*.

[7] Cura M, Haskal Z, Lopera J (2009). Diagnostic and interventional radiology for Budd-Chiari syndrome. *Radiographics*.

[8] Brancatelli G, Vilgrain V, Federle MP (2007). Budd-Chiari syndrome: spectrum of imaging findings. *American Journal of Roentgenology*.

